# Extracellular Vesicles in Osteonecrosis of the Femoral Head: An Integrated Review of Experimental and Bioinformatic Evidence

**DOI:** 10.3390/jpm16040208

**Published:** 2026-04-07

**Authors:** Elvira Immacolata Parrotta, Giorgia Lucia Benedetto, Giovanni Cuda, Umile Giuseppe Longo, Arianna Carnevale, Olimpio Galasso, Giorgio Gasparini, Michele Mercurio

**Affiliations:** 1 Department of Medical and Surgical Sciences, “Magna Græcia” University, 88100 Catanzaro, Italy; parrotta@unicz.it; 2 Department of Experimental and Clinical Medicine, “Magna Græcia” University, 88100 Catanzaro, Italy; cuda@unicz.it; 3 Fondazione Policlinico Universitario Campus Bio-Medico, 00128 Roma, Italy; g.longo@policlinicocampus.it (U.G.L.); a.carnevale@policlinicocampus.it (A.C.); 4 Research Unit of Orthopaedic and Trauma Surgery, Department of Medicine and Surgery, Università Campus Bio-Medico di Roma, 00128 Rome, Italy; 5 Department of Medicine, Surgery and Dentistry, University of Salerno, 84084 Salerno, Italy; ogalasso@unisa.it; 6 Department of Orthopaedic and Trauma Surgery, “Magna Græcia” University, “Renato Dulbecco” University Hospital, 88100 Catanzaro, Italy; gasparini@unicz.it (G.G.); michele.mercurio@unicz.it (M.M.); 7 Research Center on Musculoskeletal Health, MusculoSkeletalHealth@UMG, “Magna Græcia” University, 88100 Catanzaro, Italy

**Keywords:** osteonecrosis of the femoral head, extracellular vesicles, exosomes, targeted diagnosis, targeted therapy, bone regeneration, angiogenesis, biomarkers, precision orthopedics

## Abstract

**Background/Objectives:** Osteonecrosis of the femoral head (ONFH) is a progressive condition characterized by bone necrosis, impaired vascularization, and immune dysregulation, often resulting in femoral head collapse. Effective strategies to halt disease progression are limited. Extracellular vesicles (EVs), including exosomes and microvesicles, mediate intercellular communication and influence osteogenesis, angiogenesis, and immune responses. This review summarizes current evidence on EVs in ONFH and their translational potential. **Methods:** A structured narrative review of PubMed, Scopus, Web of Science, and Cochrane Central databases was conducted, including in vitro, preclinical, and clinical studies on EVs in ONFH. Data on EV sources, molecular cargo, signaling pathways, functional effects, and translational implications were qualitatively synthesized. No pooled statistical analysis was performed because the extracted data were heterogeneous. Bioinformatic analyses such as Gene Ontology, KEGG enrichment, and protein–protein interaction networks were also summarized. **Results:** In vitro, EVs from bone marrow mesenchymal stem cells, endothelial cells, and M2 macrophages modulate osteogenic differentiation, angiogenesis, and inflammation. Preclinical studies demonstrate that EV administration reduces femoral head necrosis, improves trabecular structure, and enhances neovascularization. Clinical studies have identified EV-associated molecules (SAA1, C4A, RPS8) linked to disease stage and the risk of femoral head collapse. Bioinformatic analyses connect EV cargo to pathways regulating bone formation, vascularization, immunity, and metabolism. **Conclusions:** EVs appear to play key roles in ONFH pathogenesis and may represent promising candidates for diagnostic and therapeutic applications. However, current clinical evidence remains limited and requires validation in larger studies. Nonetheless, heterogeneity and limited clinical data require standardized, longitudinal studies to validate their translational relevance.

## 1. Introduction

Osteonecrosis of the femoral head (ONFH) is a debilitating orthopedic condition characterized by progressive death of bone tissue, leading to joint collapse, chronic pain, and significant functional impairment [1]. Its etiology is multifactorial, encompassing corticosteroid use, alcohol abuse, traumatic injury, and idiopathic causes [2,3]. Despite advances in surgical interventions and pharmacologic treatments [4], effective strategies to halt or reverse disease progression remain limited, highlighting the need for a deeper understanding of the underlying pathophysiological mechanisms and the identification of novel therapeutic targets [5,6]. The progression of ONFH is intimately linked to disruptions in the local bone microenvironment [7], including impaired osteogenesis, defective vascularization, and dysregulated immune responses [8,9]. Proper coordination between osteoblast and osteoclast activity, coupled with adequate angiogenesis, is essential for maintaining bone homeostasis and preventing [10] necrosis [11]. In addition, the immune microenvironment plays a crucial role in modulating tissue repair and inflammation, which can either exacerbate or mitigate disease progression. Extracellular vesicles (EVs), including exosomes and microvesicles, have emerged as key mediators of intercellular communication, capable of transferring bioactive molecules such as proteins, lipids, and nucleic acids between cells [12,13]. EVs are increasingly recognized for their capacity to modulate osteogenesis, angiogenesis, and immune responses, suggesting their potential as both biomarkers and therapeutic agents in bone diseases. Recent studies have demonstrated that EVs derived from bone marrow mesenchymal stem cells (BMSCs) [14,15], macrophages, and endothelial cells can influence the regenerative microenvironment of the femoral head, promoting vascular remodeling, osteoblast differentiation, and immune modulation [16,17,18]. Their biological effects are highly context-dependent and are shaped by the cellular origin of EVs, the differentiation state of donor cells, the stage of disease, and the local microenvironment. Given the growing body of evidence, a comprehensive synthesis of the current literature is warranted to clarify the roles of EVs in ONFH and to identify mechanistic pathways with translational potential [13,19]. This review aims to integrate findings from in vitro, preclinical, and clinical studies, highlighting the functional significance of EVs in osteo-vascular regulation and immunomodulation, and to discuss their prospective applications as diagnostic biomarkers and therapeutic tools in the management of ONFH.

## 2. Materials and Methods

### 2.1. Literature Search Strategy

A review of the published literature was conducted and reported according to the Preferred Reporting Items for Systematic Reviews and Meta-Analyses (PRISMA) statement (Supplementary File S1). The PubMed, Scopus, Web of Science, and Cochrane Central databases were searched to identify relevant studies published between January 2000 and June 2025. Search terms included combinations of keywords related to extracellular vesicles, exosomes, osteonecrosis of the femoral head, osteogenesis, angiogenesis, immune modulation, bone regeneration, and biomarkers. We searched for additional articles by examining the reference lists of every article included and the gray literature available at our institution.

**Figure 1:**
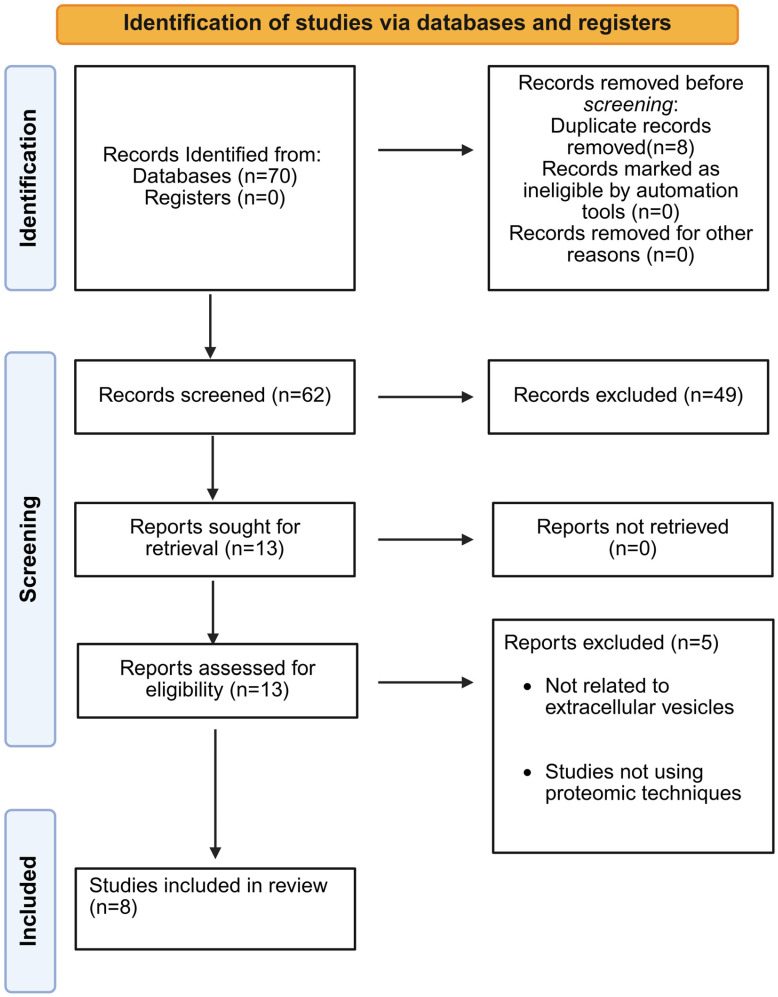
PRISMA 2020 flow diagram. The diagram summarizes the study selection process, including records identified, screened, assessed for eligibility, and ultimately included in the review based on predefined inclusion and exclusion criteria.

Only original experimental or clinical studies were included. The search strategy is reported in Supplementary File S2. The final number of studies included is reported in the PRISMA flow diagram (Figure 1).

### 2.2. Study Selection

Study selection was independently performed by two reviewers (G.L.B. and M.M.) through sequential screening of titles, abstracts, and full texts when available. Studies were included if they investigated EVs, including exosomes or microvesicles, in the context of ONFH or in biologically relevant models of bone, vasculature, or the immune system. Discrepancies between reviewers were resolved by discussion until consensus was reached [20].

### 2.3. Data Extraction and Study Categorization

For each included study, information was extracted regarding the experimental model or patient cohort, source and type of EVs, EV isolation and characterization methods, analytical techniques employed (such as proteomics, transcriptomics, metabolomics, or functional assays), and the main molecular or biological outcomes. Particular attention was given to EV-associated mechanisms related to osteogenesis, angiogenesis, immune regulation, and cellular metabolism. To facilitate interpretation and comparison across studies, the evidence was organized into three categories reflecting increasing translational relevance:

- In vitro studies exploring cellular mechanisms of EV-mediated regulation;
- Preclinical in vivo studies evaluating functional and regenerative effects of EVs in ONFH models;
- Clinical studies assessing EV cargo as potential biomarkers or translational indicators of disease progression.

No pooled statistical analysis was performed because the extracted data were heterogeneous.

### 2.4. Bioinformatic Analyses

All bioinformatic analyses summarized in this review were derived from previously published datasets and were re-integrated for interpretative purposes; no novel primary omics analyses were performed. In studies reporting molecular profiling of EV cargo, Gene Ontology (GO) enrichment analysis was used to identify overrepresented biological processes, molecular functions, and cellular components (Supplementary File S3). Functional clustering analysis, primarily using Metascape (v3.5.20260201), was considered to group enriched GO terms into interconnected biological modules. KEGG pathway enrichment was evaluated to link EV-associated molecules to canonical signaling pathways involved in bone regeneration, angiogenesis, immune modulation, and metabolism (Supplementary File S4). Protein-protein interaction (PPI) networks were constructed using the STRING database (version 12.0) to identify highly connected nodes and potential regulatory hubs. The network included proteins identified in the selected studies as well as additional interacting partners predicted by STRING (version 12.0) (Supplementary File S5). When available, disease-gene association (DISEASES) analyses were reviewed to assess the clinical relevance of EV-derived molecules in ONFH and related pathological conditions (Supplementary File S6). For this review, functional enrichment analyses were performed on proteins reported as significant in the included studies, supplemented with additional interacting proteins predicted using the STRING database (version 12.0); as full gene/protein lists were not available for all studies, these analyses are considered exploratory.

## 3. Results

The main characteristics of all included studies, including their models and EV sources, are reported in Table 1.

### 3.1. In Vitro Studies

Xiang et al. (2025) [21] reported that EVs from adipogenic and endothelial BMSCs inhibited endothelial migration and angiogenesis through modulation of the SPRY4/PTPRB/TIE2/PI3K-AKT pathway, suggesting a potential role in vascular remodeling within the femoral head. Notably, these effects on endothelial behavior may appear discordant with other reports describing pro-angiogenic EV activity. However, such differences likely reflect the strong context dependency of EV-mediated signaling, influenced by the cellular origin of EVs, the differentiation state of donor cells, and the pathological microenvironment. Rather than exerting pro- or anti-angiogenic effects, EVs appear to fine-tune vascular remodeling in a context-specific manner. Complementing these findings, Wang et al. (2022) [23] demonstrated that EVs from osteogenically differentiated BMSCs enhanced osteogenic differentiation while simultaneously balancing adipogenesis, mediated by markers such as RUNX2, ALP, COL1A1, and PPARγ,. These results indicate that BMSC-derived EVs not only promote osteoblast differentiation but also transmit pro-regenerative signals capable of counteracting early bone degeneration. Endothelial cell-derived EVs similarly contribute to vascular protection. Ma (2022) [26] observed that EVs derived from BMSCs mitigated glucocorticoid-induced injury in BMECs by activating PI3K/AKT/mTOR signaling and autophagy pathways, highlighting their protective role under stress conditions. Furthermore, EVs released from M2 macrophages appear to influence the local immune environment. Liu (2025) [27] found that these EVs modulate neutrophil extracellular trap (NET) formation and induce endothelial phenotype transitions, fostering an anti-inflammatory microenvironment that may support tissue repair. Together, these in vitro studies suggest that EVs act through integrated mechanisms, promoting osteogenesis, regulating vascularization, and modulating inflammation, highlighting their multifaceted therapeutic potential (Table 2).

### 3.2. Preclinical Studies

The therapeutic potential of EVs is further supported by evidence from animal models of ONFH. BMSC-derived EVs, for example, have been shown to activate the SPRY4/PTPRB/PI3K/AKT pathway within the femoral head, enhancing osteogenesis and angiogenesis while simultaneously reducing necrotic progression [21]. Similarly, M2 macrophage-derived EVs containing miR-93-5pand VEGF were observed to improve vascularization, attenuate inflammation, and prevent femoral head collapse [27], indicating that EVs can coordinate both vascular and immunomodulatory processes in vivo. Metabolomic analyses further support these functional observations. Guo and Zhang (2023) [28] identified significant alterations in riboflavin metabolism, CoA/pantothenate biosynthesis, glycerophospholipid metabolism, and sphingolipid metabolism in bone-derived EVs, providing mechanistic insight into how EVs may influence local cellular metabolism during ONFH progression. Histological evaluations in these models revealed improved trabecular continuity, increased bone mineral density, and enhanced neovascularization, as evidenced by upregulation of markers such as CD31, and VEGF. These findings collectively suggest that EVs exert multimodal effects, integrating regenerative, vascular, and immunomodulatory pathways to counteract ONFH pathology (Table 3). Overall, animal studies indicate that EV-based interventions exert protective effects in ONFH through the coordinated regulation of osteogenesis, angiogenesis, immune responses, and metabolic pathways. BMSC-derived EVs predominantly promote trabecular preservation and microvascular repair, whereas M2 macrophage-derived EVs further contribute immunomodulatory benefits by attenuating inflammatory signaling and NET formation. Importantly, metabolomic profiling suggests that EV-mediated regeneration is accompanied by local metabolic reprogramming, highlighting an underexplored but potentially critical component of disease modulation in vivo.

### 3.3. Clinical Studies

Clinical investigations have begun to translate preclinical findings into human contexts, highlighting the diagnostic and prognostic potential of EVs in ONFH. Serum EVs and tissue-derived exosomes from patients exhibit distinct molecular signatures that reflect disease progression and underlying pathological mechanisms. Sung et al. (2024) [22] analyzed serum EVs from patients with ONFH compared to healthy controls and identified IGHV3-23, FN1, VWF, FGB, PRG4, FCGBP, and ZSWIM9 as potential early biomarkers associated with coagulation and ECM regulation. Zhu et al. (2020) [24] examined femoral tissue-derived exosomes from patients with ONFH versus normal tissue, reporting altered expression of CD41, RUNX2, ALP, and COL1. These changes were associated with impaired osteogenic differentiation and migration, as well as modulation of focal adhesion and integrin signaling pathways. Xu et al. (2025) [25] focused on serum EVs in early versus collapsed ONFH patients, finding SAA1, C4A, and RPS8 as candidate biomarkers associated with the risk of femoral head collapse, with additional correlations to immune regulation. These findings collectively indicate that EV cargo reflects key pathological alterations in ONFH, including dysregulated coagulation, impaired osteogenesis, extracellular matrix remodeling, and immune activation. The detailed results are summarized in Table 4.

### 3.4. Integrated Bioinformatic Analysis

**Figure 2:**
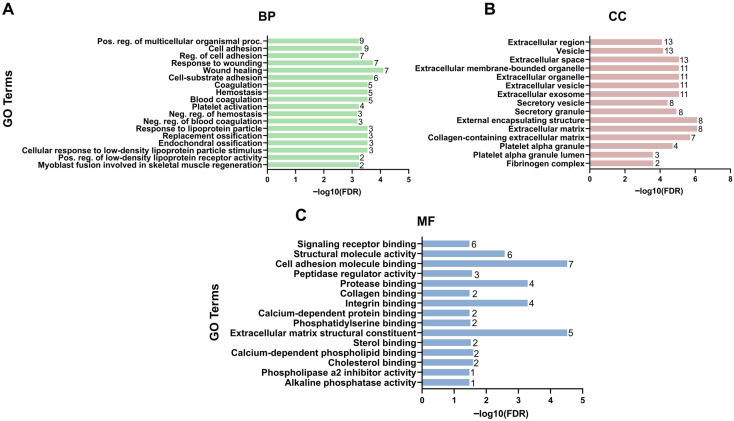
Gene Ontology enrichment analysis of EV cargo in ONFH. GO enrichment analysis of EV cargo associated with ONFH. (**A**) Enriched biological processes (BP); (**B**) Enriched cellular components (CC); (**C**) Enriched molecular functions (MF). Bars represent fold enrichment, with the length of each bar indicating the enrichment value and the number of genes shown on each bar. The *y*-axis shows the GO terms. Color indicates the −log10 false discovery rate (FDR). GO enrichment analysis of EV cargo associated with ONFH included proteins identified in the included studies and additional interacting proteins predicted using STRING (version 12.0).

To characterize the functional annotation of EV cargo in ONFH, GO enrichment analysis was performed across studies reporting molecular profiling of EV-associated proteins and metabolites. The analysis included both proteins identified in the studies and additional interacting proteins predicted using STRING (version 12.0); therefore, some proteins contributing to enriched GO terms may not have been directly observed in the original studies. Enriched biological processes included terms related to cell adhesion, coagulation, hemostasis, and myoblast fusion involved in skeletal muscle regeneration. At the level of molecular function, enriched terms involved extracellular matrix structural constituent, protease binding, cell adhesion molecule binding, and structural molecule activity. Cellular component analysis indicated enrichment of proteins associated with extracellular vesicles, extracellular matrix, fibrinogen complex, and extracellular membrane-bounded organelle (Figure 2).

**Figure 3:**
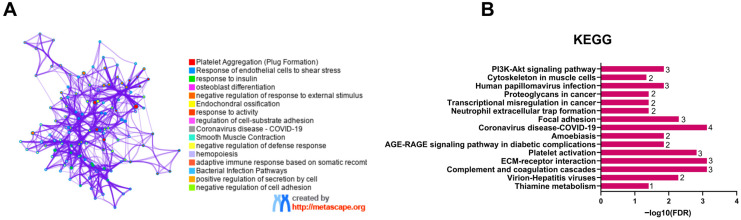
Functional clustering and KEGG pathway enrichment of EV-associated molecules in ONFH. (**A**) Metascape-based functional clustering (v3.5.20260201) showing enriched functional modules. (**B**) Bars represent fold enrichment, with the length of each bar indicating the enrichment value and the number of genes shown on each bar. The *y*-axis shows the KEGG pathways. Color indicates the −log10 false discovery rate (FDR).

Functional clustering analysis using Metascape grouped enriched GO terms into interconnected biological modules related to osteoblast differentiation, response of endothelial cells to shear stress, and response to activity. These modules showed partial overlap, indicating shared molecular components across functional categories (Figure 3A). KEGG pathway enrichment analysis mapped EV-associated molecules to canonical signaling pathways, including PI3K-AKT, cytoskeleton in muscle cells, ECM-receptor interaction, complement and coagulation cascades, and thiamine metabolism (Figure 3B).

**Figure 4:**
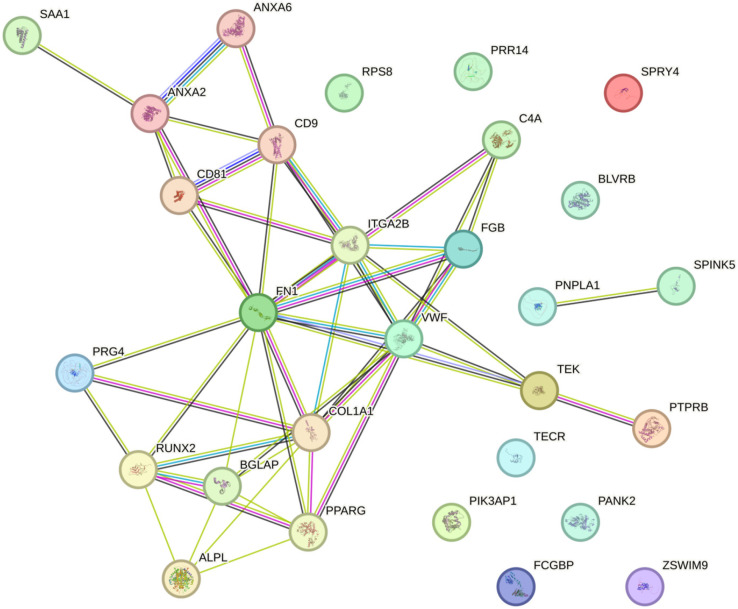
PPI network of EV-derived proteins associated with ONFH. Nodes represent EV-derived proteins identified across in vitro, preclinical, and clinical studies, while edges indicate known or predicted protein–protein interactions. The PPI network includes both proteins identified in the included studies and additional interacting proteins predicted using the STRING database (version 12.0).

PPI network analysis showed that EV-associated proteins formed interconnected interaction networks. Several highly connected nodes were identified within clusters related to extracellular matrix organization, immune response, oxidative stress, and metabolic processes (Figure 4).

**Figure 5:**
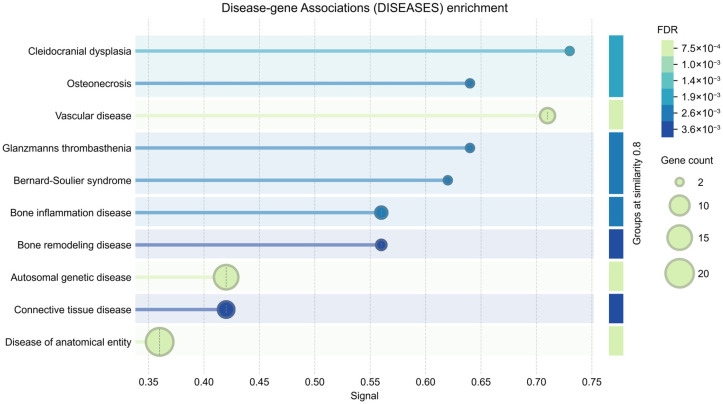
Disease-gene association (DISEASES) enrichment analysis of EV cargo. Disease-gene association analysis showing enrichment of clinically relevant disease categories associated with EV-derived proteins. Dot size represents the number of genes associated with each disease term, while color indicates the false discovery rate (FDR). The *x*-axis shows the enrichment signal. EV cargo is significantly associated with osteonecrosis, vascular diseases, inflammatory conditions, and connective tissue disorders, supporting the translational relevance of EV-mediated signaling in ONFH.

DISEASES analysis identified associations between EV-derived proteins and disease categories, including osteonecrosis, vascular disorders, inflammatory diseases, and connective tissue conditions (Figure 5).

The main findings of these integrated analyses are summarized in Table 5, showing proteins, pathways, or metabolites, the corresponding study and model, and their observed outcome or function.

## 4. Discussion

Extracellular vesicles (EVs) have emerged as multifaceted regulators in the pathophysiology of osteonecrosis of the femoral head (ONFH), operating at the intersection of osteogenesis, angiogenesis, immune modulation, and metabolic regulation. Growing evidence indicates that EVs do not function as isolated effectors but rather as integrated signaling units that coordinate multiple biological processes through the transfer of proteins, microRNAs, metabolites, and other bioactive molecules [5,13,18,29]. In the current review, the integrated bioinformatic analyses delineate a coherent molecular landscape in which EV cargo converges on four major regulatory domains: extracellular matrix organization, osteogenic differentiation, angiogenic activation, and immune/metabolic modulation. Across GO, KEGG, Metascape, and PPI datasets, recurring hubs such as RUNX2, COL1A1, VEGFA, ALP, SAA1, and components of PI3K-AKT, HIF-1, and TGF-β signaling emerge as central drivers of the EV-mediated response in ONFH. The overlap between osteogenic, vascular, inflammatory, and metabolic clusters demonstrates that EVs do not act through isolated pathways but through integrated, multilayered networks that reflect the complex pathophysiology of ONFH. Notably, the enrichment of metabolic pathways, including riboflavin, CoA, fatty acid, and sphingolipid metabolism, expands the traditional view of EV function by suggesting a substantial role in cellular energy regulation and lipid remodeling within necrotic bone.

### 4.1. EV-Mediated Regulation of Osteogenesis and Bone Remodeling

Substantial evidence supports a role for EVs in modulating osteogenic balance in ONFH. EVs derived from BMSCs and osteogenic cells carry regulatory molecules involved in osteoblast differentiation, extracellular matrix organization, and mineralization, including RUNX2, ALP, COL1A1, and associated signaling networks [18,23]. In preclinical models, administration of BMSC-derived EVs improves trabecular architecture and bone mineral density, suggesting a capacity to counteract osteogenic impairment and excessive adipogenic differentiation characteristic of early ONFH stages [21]. Notably, EV-mediated osteogenic regulation appears tightly coupled to vascular and immune pathways rather than acting as an isolated process. This integrated regulation may explain why EVs can influence mesenchymal progenitor fate decisions under pathological conditions such as glucocorticoid exposure or hypoxia, contributing to fine-tuning of bone remodeling rather than direct induction of bone formation.

### 4.2. Context-Dependent Effects on Angiogenesis

The role of EVs in angiogenesis represents one of the most debated aspects of their involvement in ONFH. Multiple studies report pro-angiogenic effects mediated through PI3K-AKT, VEGF, HIF-1, and TGF-β, signaling pathways, leading to enhanced endothelial migration, tube formation, and neovascularization in preclinical models [21,26]. Conversely, other reports describe inhibitory or modulatory effects on angiogenic responses, particularly in EVs derived from adipogenic or dysfunctional cellular sources. Rather than reflecting true contradictions, these findings likely mirror stage-specific and microenvironment-dependent requirements for vascular remodeling during ONFH progression. In early disease phases, excessive angiogenesis may exacerbate edema, intraosseous pressure, and tissue damage, whereas controlled angiogenesis becomes essential during reparative stages to restore perfusion and support osteogenesis. EVs may therefore function as modulators of vascular remodeling, adapting angiogenic responses to local pathological demands rather than acting as constitutive pro-angiogenic stimuli.

### 4.3. Immunomodulatory and Inflammatory Regulation

Beyond osteogenic and vascular effects, EVs exert significant immunomodulatory functions that are increasingly recognized as relevant to ONFH pathogenesis. EVs derived from immune cells, particularly M2 macrophages, modulate inflammatory signaling, neutrophil extracellular trap (NET) formation, and endothelial phenotype transitions, contributing to the regulation of inflammation [27]. These immunomodulatory effects intersect with vascular and osteogenic pathways, reinforcing the concept that immune, endothelial, and skeletal compartments are functionally interconnected in ONFH. By shaping local inflammatory tone, EVs may indirectly influence osteoblast survival, progenitor recruitment, and matrix remodeling.

### 4.4. Emerging Metabolic Dimension of EV Function

An important and relatively underexplored insight emerging from recent studies is the involvement of EVs in metabolic regulation during ONFH. Metabolomic analyses of bone-derived EVs reveal alterations in riboflavin metabolism, pantothenate and coenzyme A biosynthesis, glycerophospholipid metabolism, and sphingolipid pathways [28]. These findings suggest that EVs may contribute to cellular energy homeostasis, redox balance, and lipid remodeling under hypoxic and metabolically stressed conditions. Given that metabolic dysfunction could represent a hallmark of ONFH, particularly in steroid-induced and metabolic forms of the disease, EV-mediated metabolic reprogramming may constitute a critical but underappreciated component of tissue adaptation and repair.

### 4.5. Translational Relevance and Biomarker Potential

**Figure 6:**
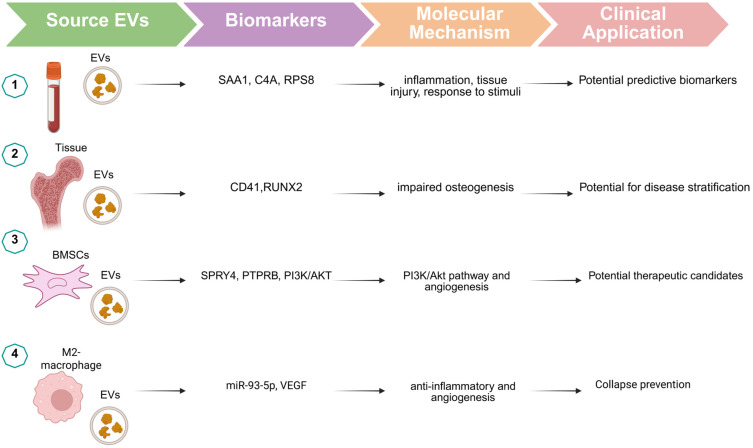
Clinical translation framework of EVs in ONFH. Schematic overview of EV-mediated translational pathways in ONFH, linking EV sources, molecular cargo identified in experimental and clinical studies, downstream biological mechanisms, and potential diagnostic and therapeutic applications. Numbers 1-4 indicate different EV sources: (1) blood-derived EVs, (2) tissue-derived EVs, (3) bone marrow mesenchymal stem cell (BMSC)-derived EVs, and (4) M2 macrophage-derived EVs.

Clinical investigations, although still limited, suggest potential translational relevance of EV cargo in ONFH. Proteomic profiling of circulating EVs from ONFH patients has identified molecules such as SAA1, C4A, and RPS8, reflecting inflammatory activation and metabolic stress, while osteogenic markers, including RUNX2 and ALP, emerge from preclinical EV studies investigating impaired bone regeneration [22]. These molecular signatures parallel observations from preclinical models and support the potential of EVs as minimally invasive biomarkers for disease staging and progression (Figure 6, Table 6) [22,30].

However, while these associations are biologically plausible and clinically appealing, current evidence does not yet establish EVs as validated diagnostic or prognostic tools [31]. Most available studies involve small cohorts, cross-sectional designs, and heterogeneous methodologies, limiting reproducibility and clinical generalizability. In addition to their biomarker potential, EVs may represent a promising platform for stage-oriented therapeutic strategies; however, current evidence remains largely preclinical. Their intrinsic biocompatibility, low immunogenicity, and natural capacity to transfer osteogenic, angiogenic, immunomodulatory, and metabolic signals support their possible development as multimodal delivery systems. In early-stage ONFH, predominantly characterized by vascular impairment and cellular stress without structural collapse, EVs enriched in angiogenic and cytoprotective cargo could theoretically contribute to microvascular restoration and osteoblast survival, potentially complementing joint-preserving approaches. During reparative phases, EV-mediated enhancement of osteogenic differentiation and controlled angiogenesis may improve trabecular remodeling and optimize the biological environment following procedures such as core decompression. In contrast, in advanced stages with established femoral head collapse, EV-based interventions are unlikely to reverse structural damage but may serve as adjuvant strategies aimed at modulating inflammation and supporting bone remodeling in conjunction with surgical management. Emerging bioengineering approaches, including donor cell preconditioning, EV surface modification to enhance bone targeting, and incorporation into biomaterial scaffolds, further suggest that EVs could evolve from endogenous mediators to customizable therapeutic carriers. In line with this concept, stem cell-derived EVs have been increasingly explored as cell-free therapeutic platforms in musculoskeletal disorders, as their molecular cargo can modulate regenerative and inflammatory pathways involved in tissue repair and skeletal homeostasis [32]. Although these strategies remain largely preclinical, integrating EV profiling with radiological staging systems may provide a more biologically informed framework for patient stratification and precision-oriented management in ONFH.

### 4.6. Limitations and Future Directions

Despite promising advances, several limitations constrain the immediate clinical translation of EV-based strategies in ONFH. First, significant heterogeneity persists in EV sources, isolation methods, dosing regimens, and molecular profiling platforms, complicating cross-study comparisons and reproducibility. Preclinical models often fail to capture the etiological and clinical heterogeneity of human ONFH, including steroid-induced, alcohol-related, metabolic, and idiopathic forms. Second, this search involved four major literature databases, so we cannot exclude the possibility that additional articles could have been identified using other databases. In addition, the limited cohort sizes in existing clinical studies reflect the current early stage of research in EVs and ONFH, with all available studies meeting inclusion criteria being considered. Moreover, clinical studies remain scarce, typically underpowered, and lack standardized outcome measures and longitudinal follow-up. Additional challenges include incomplete characterization of EV biodistribution, safety, and optimal delivery strategies. In this context, future research should also explore EV profiling as a tool for targeted diagnosis and patient stratification, moving beyond purely radiological staging. Similarly, EV-based interventions may represent a framework for mechanism-guided and personalized therapeutic strategies, provided that robust standardization and clinical validation are achieved. To advance the field, future research should prioritize standardized methodological frameworks, etiology- and stage-specific analyses, and rigorously designed translational and clinical studies. Similarly, EV-based interventions may represent a framework for mechanism-guided and personalized therapeutic strategies, provided that robust standardization and clinical validation are achieved [33].

Furthermore, while the present study focuses on ONFH-derived EVs, comparative analyses across related musculoskeletal conditions such as osteoarthritis and osteoporosis, including KEGG pathway enrichment and molecular profiling, could provide additional insights into shared and disease-specific EV signatures. Although integrating such heterogeneous datasets is beyond the scope of the current work, future studies are encouraged to pursue this direction to enhance mechanistic understanding and translational relevance [33].

## 5. Conclusions

The current review showed that extracellular vesicles represent a compelling avenue for understanding ONFH and exploring future diagnostic and therapeutic applications. By unifying experimental, clinical, and bioinformatic data, this review uniquely delineates a stage-oriented mechanistic framework of EVs in ONFH, offering a translational perspective for future diagnostic and therapeutic strategies. Across GO, KEGG, Metascape, and PPI datasets, recurring hubs such as RUNX2, COL1A1, VEGFA, ALP, SAA1, and components of PI3K-Akt, HIF-1, and TGF-β signaling emerged as central drivers of the EV-mediated response. The unique capacity of EVs to integrate osteogenic, angiogenic, immunomodulatory, and metabolic signals could be used for a multitarget approach. While EV-based diagnostics and therapies remain investigational, continued research supported by standardized protocols, scalable production strategies, and well-designed longitudinal clinical studies will be essential to determine whether EVs can be safely and effectively integrated into stage-specific management algorithms and, potentially, to modify the natural history of ONFH.

## Figures and Tables

**Table 1:** Overview of studies investigating EVs in ONFH.

Author	Population/Model	EVs	Technique
Xiang et al., 2025 [21]	In vitro and SD rat	Exosomes	RNA-seq, mass spectrometry, WB
Sung et al., 2024 [22]	ONFH patients *n* = 11 vs. healthy *n* = 11	Serum-derived EVs	LC-MS/MS + ELISA
Wang et al., 2022 [23]	In vitro BMSC cultures	EVs from differentiated BMSCs	LC-MS/MS + WB
Zhu et al., 2020 [24]	ONFH patients *n* = 30 vs. healthy *n* = 30	Tissue-isolated exosomes	LC-MS/MS + WB
Xu et al., 2025 [25]	Early ONFH patients *n* = 11 vs. collapsed *n* = 9	Serum-derived exosomes	LC-MS/MS
Ma et al., 2022 [26]	In vitro	EVs from BMSCs	Functional assays, pathway analysis
Liu et al., 2025 [27]	In vitro/animal ONFH models	Exosomes	Functional assays
Guo, M., Zhang, J, 2023 [28]	ONFH patients *n* = 30 vs. femoral neck fracture *n* = 30	Exosomes	UPLC-MS/MS metabolomics

Summary of studies investigating extracellular vesicles (EVs) in ONFH. The table reports authors/year, experimental model or patient cohort, EV source/type, and analytical techniques. Abbreviations: BMSC, bone marrow mesenchymal stem cell; EVs, extracellular vesicles; ONFH, osteonecrosis of the femoral head; LC-MS/MS, liquid chromatography-tandem mass spectrometry; UPLC-MS/MS, ultra-performance liquid chromatography-tandem mass spectrometry; WB, Western blot.

**Table 2:** In vitro effects of extracellular vesicles on cellular models relevant to ONFH.

Study	Model/Cells	Treatment	Comparison	Biomarkers	Main Findings
Xiang et al., 2025 [21]	Adipogenic BMSCs and endothelial cells	Exosomes	Adipogenic BMSC vs. control exosomes	SPRY4, PTPRB, TIE2, PI3K/AKT	Inhibition of endothelial migration, impaired angiogenesis, and downregulated angiogenic gene expression
Wang et al., 2022 [23]	Osteogenically differentiated BMSCs	EVs from differentiated BMSCs	EVs from differentiated BMSCs vs. naïve	CD9, CD81, COL1A1, RUNX2, ALP, PPARγ	Regulation of osteogenic vs. adipogenic differentiation; support for bone regeneration
Ma et al., 2022 [26]	BMECs treated with glucocorticoids	EVs from BMSCs	Glucocorticoid vs. control	PI3K/AKT/mTOR, autophagy	Prevention of glucocorticoid-induced injury via autophagy regulation
Liu et al., 2025 [27]	M2 macrophages	Exosomes	Treatment vs. control	NETs, endothelial phenotype	Modulation of NETs formation and endothelial phenotype: potential therapeutic effect

This table summarizes in vitro studies investigating the effects of EVs on cell types relevant to ONFH. For each study, the cell model, type and source of EVs, key molecular markers analyzed, and main findings are reported. The findings encompass effects on osteogenesis, angiogenesis, and immune modulation. Abbreviations: BMSCs: Bone marrow mesenchymal stem cells; BMECs: Bone microvascular endothelial cells; EVs: Extracellular vesicles; NETs: Neutrophil extracellular traps; ALP: Alkaline phosphatase; COL1A1: Collagen type I alpha 1 chain; PPARγ: Peroxisome proliferator-activated receptor gamma; RUNX2: Runt-related transcription factor 2; PI3K/AKT: Phosphatidylinositol 3-kinase/Protein kinase B; TIE2: TEK receptor tyrosine kinase mTOR: Mechanistic target of rapamycin kinase.

**Table 3:** In vivo effects of extracellular vesicles in animal models of ONFH.

Study	Model/Tissue	Treatment	Comparison	Biomarkers	Main Findings
Xiang et al., 2025 [21]	SD rat, adipogenic BMSCs, and endothelial cells	Exosomes	Adipogenic BMSC vs. control exosomes	SPRY4, PTPRB, TIE2, PI3K/AKT	Modulation of angiogenesis and endothelial migration
Liu et al., 2025 [27]	ONFH animal models	Exosomes	Treatment vs. control	NETs, endothelial phenotype	Modulation of NETs formation and endothelial phenotype
Guo, M., Zhang, J, 2023 [28]	ONFH models	Exosomes	ONFH vs. control	Metabolites: riboflavin, CoA, phospholipids, sphingolipids	Identification of metabolic signatures and mechanistic insights in ONFH

This table summarizes in vivo studies investigating the effects of EVs in animal models relevant to ONFH. For each study, the animal model or tissue used, the type of EV treatment, key molecular or metabolic markers analyzed, and the main findings are reported. The findings highlight effects on angiogenesis, endothelial function, immune modulation, and metabolic signaling in ONFH. Abbreviations: BMSCs: Bone marrow mesenchymal stem cells; NETs: Neutrophil extracellular traps; SD rat: Sprague-Dawley rat; CoA: Coenzyme A.

**Table 4:** Clinical findings of extracellular vesicles in ONFH.

Study	Sample Type	Comparison	Biomarkers	Main Findings
Sung et al., 2024 [22]	Serum-derived EVs	ONFH vs. healthy	IGHV3-23, FN1, VWF, FGB, PRG4, FCGBP, ZSWIM9	Potential early biomarkers related to coagulation and ECM
Zhu et al., 2020 [24]	Femoral tissue-isolated exosomes	ONFH vs. normal	CD41, RUNX2, ALP, COL1	Impaired osteogenic differentiation and migration; focal adhesion and integrin signaling modulation
Xu et al., 2025 [25]	Serum-derived exosomes	Early ONFH vs. collapsed stage	SAA1, C4A, RPS8	Potential predictive biomarkers for femoral head collapse: correlation with immune regulation

This table summarizes clinical studies investigating EVs in ONFH, reporting sample type, comparison group, key biomarkers, and main findings related to disease progression, osteogenic potential, coagulation, extracellular matrix remodeling, and immune regulation. Abbreviations: EVs: Extracellular vesicles; ALP: Alkaline phosphatase; RUNX2: Runt-related transcription factor 2; IGHV3-23: Immunoglobulin heavy variable 3-23; FN1: Fibronectin 1; VWF: von Willebrand factor; FGB: Fibrinogen beta chain; PRG4: Proteoglycan 4; FCGBP: Fc fragment of IgG binding protein; ZSWIM9: Zinc finger SWIM-type containing 9; SAA1: Serum amyloid A1; C4A: Complement C4A; RPS8: Ribosomal protein S8.

**Table 5:** Key EV-derived proteins, pathways, and metabolites identified in bioinformatic analyses.

Protein/Pathway/Metabolite	Study	Model	Outcome/Function
SPRY4, PTPRB, TIE2, PI3K/AKT	Xiang et al., 2025 [21]	Adipogenic and endothelial BMSCs, femoral bone tissue	Inhibition of endothelial migration and angiogenesis; modulation of PTPRB/PI3K/AKT
IGHV3-23, FN1, VWF, FGB, PRG4, FCGBP, ZSWIM9	Sung et al., 2024 [22]	Serum	Potential early biomarkers for ONFH, related to coagulation and ECM
CD9, CD81, COL1A1, ALP, RUNX2, PPARγ	Wang et al., 2022 [23]	Osteogenically differentiated BMSCs	Regulation of osteogenic vs. adipogenic differentiation; support for bone regeneration
CD41, RUNX2, ALP, COL1,	Zhu et al., 2020 [24]	Femoral tissue	Impaired osteogenic differentiation and migration; modulation of focal adhesion and integrin signaling
SAA1, C4A, RPS8	Xu et al., 2025 [25]	Serum	Potential predictive biomarkers for femoral head collapse; correlation with immune regulation
PI3K/Akt/mTOR, autophagy	Ma et al., 2022 [26]	BMECs treated with glucocorticoids	Prevention of glucocorticoid-induced injury via autophagy regulation
NETs, endothelial phenotype transition	Liu et al., 2025 [27]	M2 macrophages	Modulation of NET formation and endothelial phenotype: potential therapeutic effect
Differential metabolites/pathways: Riboflavin metabolism, Pantothenate and CoA biosynthesis, Glycerophospholipid metabolism, and Sphingolipid metabolism	Guo, M., Zhang, J, 2023 [28]	Bone-derived exosomes	Identification of metabolite signatures and mechanistic insights in ONFH

This table summarizes key proteins, pathways, and metabolites carried by EVs across different studies and models. For each entry, the study, experimental model, and observed functional outcome are reported, highlighting their role in osteogenesis, angiogenesis, immune modulation, and metabolic regulation in ONFH. Abbreviations: Bone marrow mesenchymal stem cells; BMECs: Bone microvascular endothelial cells; ECM: Extracellular matrix; PI3K/AKT: Phosphatidylinositol 3-kinase/Protein kinase B; mTOR: Mechanistic target of rapamycin kinase; NETs: Neutrophil extracellular traps; ALP: Alkaline phosphatase; COL1A1: Collagen type I alpha 1 chain; RUNX2: Runt-related transcription factor 2; PPARγ: Peroxisome proliferator-activated receptor gamma.

**Table 6:** Translational relevance of extracellular vesicles in ONFH.

EV Source	Key Biomarkers/Cargo	Cohort/Model	Sample	Translational Insight
Serum EVs	SAA1, C4A, RPS8	Early vs. collapsed ONFH patients	Serum	Potential predictive biomarkers for femoral head collapse
Tissue exosomes	CD41 deficiency, integrin β3-FAK-AKT-RUNX2 pathway	ONFH patients vs. controls	Femoral head tissue	Indicators of impaired osteogenic potential
BMSC-derived EVs	SPRY4, PTPRB, TIE2/PI3K/AKT	Rat ONFH model	Bone tissue	Impaired angiogenesis and osteonecrosis progression
M2 macrophage EVs	miR-93-5p, VEGF	Steroid-induced ONFH rats	Bone/endothelial cells	Prevent collapse and enhance vascularization

This table summarizes the translational potential of EVs in ONFH. EVs from different sources demonstrate predictive, regenerative, and angiogenic effects in both patients and preclinical models, highlighting their role as diagnostic indicators and potential therapeutic agents. Abbreviations: EVs: Extracellular vesicles; RUNX2: Runt-related transcription factor 2; PI3K/AKT: Phosphatidylinositol 3-kinase/Protein kinase B; VEGF: Vascular endothelial growth factor; SAA1: Serum amyloid A1; C4A: Complement C4A; RPS8: Ribosomal protein S8.

## Data Availability

All the data generated or analyzed during this study are included in this published article and its Supplementary Information Files.

## Supplementary Materials

The following supporting information can be downloaded at https://www.mdpi.com/article/10.3390/jpm16040208/s1: Supplementary File S1: Prisma Checklist [34]; Supplementary File S2. Full search strategy for PubMed, Scopus, Web of Science, and Cochrane Central; Supplementary File S3. Gene Ontology enrichment analysis results (ShinyGO (version 0.85.1) output, .xlsx format); Supplementary File S4: KEGG enrichment analysis results (ShinyGO (version 0.85.1) output, .xlsx format); Supplementary File S5. Protein–protein interaction network table exported from STRING (tsv. format); Supplementary File S6. Disease-gene association (DISEASES) enrichment analysis from STRING (tsv format).

## Abbreviations

The following abbreviations are used in this manuscript:

BMSCs	Bone Marrow Mesenchymal Stem Cells
BMECs	Bone microvascular Endothelial Cells
CoA	Coenzyme A
DISEASES	Disease-gene association enrichment
ECM	Extracellular Matrix
EVs	Extracellular Vesicles
GO	Gene Ontology
HIF-1	Hypoxia-Inducible Factor 1
LC–MS/MS	Liquid Chromatography-Tandem Mass Spectrometry
mTOR	Mechanistic Target of Rapamycin Kinase
NETs	Neutrophil Extracellular Traps
ONFH	Osteonecrosis of the Femoral Head
PI3K/AKT	Phosphatidylinositol 3-Kinase/Protein Kinase B
UPLC-MS/MS	Ultra-Performance Liquid Chromatography- Tandem Mass Spectrometry
VEGF	Vascular Endothelial Growth Factor
WB	Western Blot
