# Supplementary material for: Extracellular Vesicles in Osteonecrosis of the Femoral Head: An Integrated Review of Experimental and Bioinformatic Evidence
Source: J Pers Med. 2026 Apr 7;16(4):208. doi: 10.3390/jpm16040208 (PMC13117771; doi:10.3390/jpm16040208)
Supplement: Supplementary file 1 [file jpm-16-00208-s001.zip › Supplementary File 2.pdf]

Full search strategy for PubMed, Scopus, Web of Science, and Cochrane Central Query

SCOPUS

1. TITLE-ABS-KEY(("osteonecrosis" OR "avascular necrosis") AND ("extracellular vesicle" OR exosome OR "microvesicle") AND (proteomic OR "mass spectrometry"));
2. TITLE-ABS-KEY (("osteonecrosis" OR "avascular necrosis") AND exosome AND (serum OR plasma) AND (proteomic OR "mass spectrometry"))

Query PUBMED

1. osteonecrosis AND extracellular vesicle AND BMSC;
2. osteonecrosis AND extracellular vesicle;
3. osteonecrosis AND extracellular vesicle AND proteomics;
4. osteonecrosis of the femoral head OR ONFH AND exosomes AND adipose stem cells;
5. osteonecrosis of the femoral head AND proteomics AND cartilage;
6. osteonecrosis of femoral head OR ONFH AND M2 macrophages-derived exosomes;
7. osteonecrosis of the femoral head AND exosome AND metabolomic;

Query Web of Science

TS=(("osteonecrosis" OR "avascular necrosis" OR "femoral head necrosis" OR ONFH) AND ("extracellular vesicle*" OR exosome* OR microvesicle*) AND ("proteomic*" OR "mass spectrometry" OR "LC-MS/MS")).

Query of Cochrane Central

("osteonecrosis" OR "avascular necrosis" OR "femoral head necrosis") AND ("extracellular vesicle" OR exosome) AND ("proteomic" OR "mass spectrometry").
